# Activation of macrophages by extracellular vesicles derived from *Babesia*-infected red blood cells

**DOI:** 10.1128/iai.00333-24

**Published:** 2025-04-02

**Authors:** Biniam Hagos, Ioana Brasov, Heather Branscome, Sujatha Rashid, Rebecca Bradford, Joseph Leonelli, Fatah Kashanchi, Choukri Ben Mamoun, Robert E. Molestina

**Affiliations:** 1 American Type Culture Collection 36576, Manassas, Virginia, USA; 2 School of Systems Biology, George Mason University 208360, Manassas, Virginia, USA; 3 Section of Infectious Disease, Yale University School of Medicine, New Haven, Connecticut, USA; Tulane University, New Orleans, Louisiana, USA

**Keywords:** *Babesia*, macrophages, extracellular vesicles, innate immunity, NF-kappa B, cytokines

## Abstract

*Babesia microti* is the primary cause of human babesiosis in North America. Despite the emergence of the disease in recent years, the pathogenesis and immune response to *B. microti* infection remain poorly understood. Studies in laboratory mice have shown a critical role for macrophages in the elimination of parasites and infected red blood cells (iRBCs). Importantly, the underlying mechanisms that activate macrophages are still unknown. Recent evidence identified the release of extracellular vesicles (EVs) from *Babesia* iRBCs. EVs are spherical particles released from cell membranes under natural or pathological conditions that have been suggested to play roles in host–pathogen interactions among diseases caused by protozoan parasites. The present study examined whether EVs released from cultured *Babesia* iRBCs could activate macrophages and alter cytokine secretion. An analysis of vesicle size in EV fractions from *Babesia* iRBCs showed diverse populations in the <100 nm size range compared to EVs from uninfected RBCs. In co-culture experiments, EVs released by *B. microti* iRBCs appeared to be associated with macrophage membranes and cytoplasm, indicating uptake of these vesicles *in vitro*. Interestingly, the incubation of macrophages with EVs isolated from *Babesia* iRBC culture supernatants resulted in the activation of NF-κB and modulation of pro-inflammatory cytokines. These results support a role for *Babesia*-derived EVs in macrophage activation and provide new insights into the mechanisms involved in the induction of the innate immune response during babesiosis.

## INTRODUCTION

Babesiosis is an emerging tick-borne disease in the United States caused by intraerythrocytic protozoan parasites of the genus *Babesia* (1). *Babesia microti* is the primary causative agent for most human cases of babesiosis. Transmission occurs primarily by Ixodes tick vectors, with less common human-to-human transmission routes including pregnancy and blood transfusion (2, 3). The latter is of concern given the fact that babesiosis is the most frequently reported transfusion-transmitted parasitic infection in the United States (4–6). Human babesiosis is usually asymptomatic in immunocompetent populations or results in mild symptoms that resolve within a few days. Immunocompromised individuals may experience severe symptoms of acute anemia, thrombocytopenia, organ failure, and even death (2, 3).

Despite the emergence of human cases of babesiosis in recent years, the pathogenesis and immune response to *B. microti* infection remain poorly understood. Splenectomized or innately asplenic mice are highly susceptible to infection, whereas infected immunocompetent mice and hamsters display significantly enlarged spleens due to increased numbers of macrophages (7, 8). Macrophage depletion results in elevated parasitemia and mortality in mice, highlighting the contribution of macrophages in the elimination of parasites and parasitized RBCs during babesiosis (9). Importantly, the effector parasite molecules that trigger the innate immune response to *B. microti* are still unknown.

Contrary to *Plasmodium,* which develops inside a parasitophorous vacuole (PV) following RBC invasion, *B. microti* forms a transient PV upon invasion, which eventually disintegrates as the parasite develops in the RBC cytoplasm (10, 11). Giemsa-stained blood smears of *B. microti*-infected RBCs show different morphological changes such as ring-shaped forms, membranous extensions protruding from rings, and the less common tetrad forms (12). The membranous extensions have been described as dendrite-like tubulovesicular structures or tubes of vesicles (TOVs) (12). These TOVs originate from the vesiculation of the *B. microti* plasma membrane, followed by the interlacement of connected vesicles within the RBC cytoplasm. It has been postulated that this system of vesicles extending from the parasite into the RBC represents a novel mechanism of protein trafficking and export (12). Importantly, the vesicle-mediated protein export is restricted not only to the host RBC but also to the extracellular environment, as shown by the detection of *B. microti* immunodominant antigens in EVs isolated from the plasma of infected mice (12, 13). A recent report by Beri et al. identified EVs released from RBCs infected *in vitro* with *B. divergens* (13).

We posit that the recently described *B. microti* protein export system is a mechanism by which parasite antigens enclosed within EVs released to the extracellular environment participate in cell-to-cell communication, similar to mechanisms reported in malaria (14–17). When macrophages function as recipients of EVs from *B. microti* iRBCs, changes in cytokine modulation with key roles in the host innate immune response to the parasite occur. To address this hypothesis, this study examined cytokine responses in macrophages exposed to EVs derived from *B. microti* iRBCs. We also assessed the diversity of vesicle populations found in iRBC-derived EV fractions by size distribution analysis and examined EV uptake by macrophages. Results from this study provide insights into the mechanisms of intercellular communication between *Babesia* and macrophages, which are plausibly critical to the induction of the innate immune response in the mammalian host.

## MATERIALS AND METHODS

### *Babesia microti* isolate

*Babesia microti* GI (ATCC PRA-398) was originally isolated from blood obtained from a human case of babesiosis in Nantucket, Massachusetts, USA, in 1983 (18, 19). The isolate was maintained by *in vivo* propagation in Syrian hamsters (Stock HsdHan:AURA, Inotiv, Indianapolis, IN) according to published protocols (20, 21) and procedures approved by the ATCC Institutional Animal Care and Use Committee.

### Short-term *in vitro* culture of *B. microti*

Blood was collected from a *B. microti*-infected hamster at 25% parasitemia, and leukocytes were depleted by passing the sample through an Acrodisc white blood cell syringe filter (Pall Biotech, Westborough, MA). Infected blood was diluted fivefold to 5% parasitemia by mixing 120 µL of hamster blood with 480 µL of leukocyte-depleted uninfected human blood (Interstate Blood Bank, Philadelphia, PA). The uninfected control consisted of 120 µL of uninfected hamster blood mixed with 480 µL of uninfected human blood. Erythrocyte cultures were established at 5% hematocrit by resuspending 600 µL of infected or uninfected blood sample in 12 mL of HL-1 medium (Lonza, Basel, Switzerland) supplemented with 20% human serum type A^+^ (Interstate Blood Bank), 1% (v/v) HB 101 (Irvine Scientific, Santa Ana, CA), 2 mM L-glutamine (ATCC, Manassas, VA), 2× hypoxanthine/thymidine solution, 1× antibiotic/antimycotic solution, and 100 µg/mL gentamicin solution (Thermo Fisher, Waltham, MA). Blood samples resuspended in growth media were transferred to 6-well plates using 2 mL of suspension per well, and erythrocyte cultures were incubated at 37°C under 2% O_2_, 5% CO_2_, and 93% N_2_ atmospheric conditions for up to 96 hours (22). Parasitemia was checked daily by microscopic examination of Giemsa-stained blood smears.

### Macrophage cultures

The THP-1 human monocytic cell line ATCC TIB-202 and the NF-κB reporter human monocytic cell line ATCC TIB-202-NFkB-LUC2 were maintained at 37°C with 5% CO_2_ in RPMI-1640 medium supplemented with 10% FBS, 100 U/mL penicillin, and 100 µg/mL streptomycin. Monocyte-derived macrophages were derived by treating the cell lines with 100 ng/mL of phorbol 12-myristate 13-acetate (PMA; Sigma, St. Louis, MO) for 48 hours, followed by a 24 hour incubation in medium alone before experiments. All the medium components were obtained from ATCC, and the cell lines were tested for the absence of bacterial contamination using BacT/ALERT (BioMérieux, Durham, NC) and mycoplasma contamination by Hoechst DNA stain, culture, and PCR.

### Isolation of EVs from RBC culture supernatants

uRBC and *B. microti* iRBC cultures were established in 6-well plates using the conditions described above. Supernatants (15 mL) were collected after 24 and 48 hours of incubation and centrifuged at 500 × *g* for 10 minutes to pellet RBCs. Samples were subsequently subjected to stepwise centrifugations at 2,000 × *g* for 10 minutes (2K), 10,000 × *g* for 40 minutes (10K), 100,000 × *g* for 90 minutes (100K), and 167,000 × *g* for 16 hours (167K) (Barclay et al., 2019; DeMarino et al., 2018). EV pellets were resuspended in 200 µL of PBS and stored at −80°C before use. In separate experiments, EVs were enriched from RBC culture supernatants using ExoMax according to the manufacturer’s instructions (System Biosciences, Palo Alto, CA). EVs were examined for protein concentration using BCA and Western blot as described below.

### Western blots

Proteins in EV samples were resolved by SDS-PAGE and transferred onto polyvinylidene difluoride (PVDF) membranes. PVDF membranes were blocked in PBS with 3% nonfat milk powder and 0.05% Tween 20 for 1 hour. Membranes were then probed with 1:500 dilutions of rabbit polyclonal antibodies raised against host–parasite antigen BmIPA48 (23). Primary antibody binding was detected by a goat-anti-rabbit antibody conjugated to horseradish peroxidase (HRP) (1:2000 dilution; Thermo Fisher). Signals were detected using a chemiluminescence substrate and the Azure c600 Imaging System (Azure Biosystems, Dublin, CA).

### Quantification of EVs and analysis of EV size during infection

The concentration (particles/mL) and diameter of EVs isolated from culture supernatants were examined by nanoparticle tracking analysis (NTA) using the Nanosight NS300 instrument (Malvern Panalytical, Westborough, MA). Each sample was analyzed in triplicate, with equipment settings remaining constant between readings. Comparisons between EV fractions obtained from RBC culture supernatants during the 24 to 48 hour time frame were performed to evaluate possible changes in the number and size distribution of EVs released during infection. Analysis of samples from uninfected RBCs served as controls to determine potential increases in EV secretion as a result of *B. microti* infection.

### NF-κB activation assay

Monocyte-derived macrophages of the ATCC TIB-202-NFkB-LUC2 cell line were cultured in 12-well plates at 2 × 10^6^ cells/well and incubated for 24 hours at 37°C, 5% CO_2_, with increasing concentrations of EVs from uRBC or *B. microti* iRBC culture supernatants. Macrophages treated with 5 µg/mL of LPS for 24 hours were used as positive controls. Following incubations, culture supernatants were frozen at −80°C, and macrophages were processed according to the Promega E1500 Luciferase Assay System protocol (Promega, Madison, WI). Cells were lysed and incubated with D-luciferin substrate for 5 minutes in white Lumitrac plates (Greiner Bio-one, Monroe, NC). Luminescence was analyzed using a SpectraMax M5 system connected to SoftMax Pro version 8.0 software (Molecular Devices, San Jose, CA).

### Cytokine arrays

Macrophage supernatants were thawed on ice and assayed for the presence of 40 cytokines using Proteome Profiler Antibody Arrays (R&D Systems, Minneapolis, MN). Procedures were followed according to the manufacturer’s instructions. Chemiluminescent signals from the arrays corresponding to the different cytokines were detected using the Azure c600 Imaging System (Azure Biosystems). Densitometric analyses of cytokine spots were performed using the ImageJ software (https://ij.imjoy.io/). Integrated densitometric values (IDV) were normalized to spots corresponding to PBS-negative controls on the arrays, and the numerical data was plotted using GraphPad Prism software version 8.0.

### Assessment of EV uptake by macrophages

EVs isolated in 167K fractions from RBC supernatants were labeled with BODIPY 493/503 (Thermo Fisher). The dye was prepared in DMSO at a concentration of 1 mM. A 5 µL of the dye stock solution was mixed with approximately 10^7^ EVs in 100 µL of PBS. The EV suspension was incubated at 37°C for 30 minutes in the dark and loaded into spin columns packed with G-10 Sephadex. Excess unincorporated dye was removed by centrifugation of the spin columns at 500 × *g* for 2 minutes. The efficiency of EV labeling and the quantification of EVs were performed by NTA as described above. Monocyte-derived macrophages of the ATCC TIB-202 cell line, cultured in 8-well chamber slides at 2 × 10^4^ cells/well, were incubated at 37°C, 5% CO_2_, for 0.5, 1, 2, and 3 hours with approximately 2 × 10^6^ labeled EVs/well. Cells were fixed in 4% paraformaldehyde for 10 minutes, washed in PBS, and visualized under 640× magnification using a Zeiss Axioscope fluorescence microscope connected to a digital camera. Digital microscopic images were captured using Zen Imaging Software (Zeiss, Oberkochen, Germany).

### Data analysis

Data from NF-κB luciferase assays and cytokine arrays were collected from three experiments. In each NF-κB experiment, luminescence measurements were performed in duplicate for each sample (Fig. 4). In each cytokine array experiment, IDV measurements were performed in duplicate spots for each cytokine. Data from these experiments were subsequently analyzed using GraphPad Prism 8 (GraphPad Software, Inc., San Diego, CA, USA) to calculate the means and standard error of the mean (SEM). Where indicated, results were subjected to analysis of variance (ANOVA), followed by Tukey’s multiple-comparison test. A *P* value of <0.05 was used to determine statistical significance.

## RESULTS

### *In vitro* culture of *B. microti*

**Fig 1:**
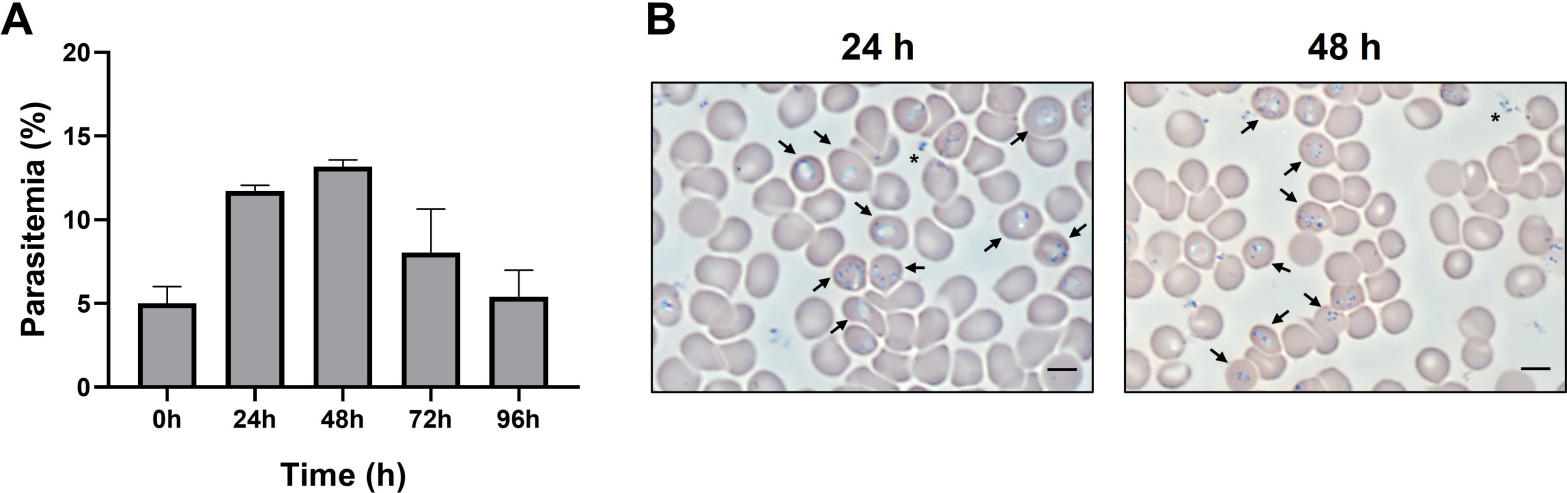
Short-term *in vitro* culture of *B. microti*. (A) Parasitemia of iRBC cultures was determined by microscopy. Columns represent means + SEM of three replicates from a representative experiment. (B) Representative image of intracellular stages of *B. microti* in iRBCs (arrows). Proteome extracellular merozoites are depicted by asterisks. Bar, 6 µm.

A continuous *in vitro* model system for *B. microti* is currently unavailable; however, small-scale studies are possible using short-term cultures. In our studies, blood was collected from *B. microti*-infected hamsters at 25% parasitemia and diluted to 5% parasitemia with uninfected human blood at a 50% hematocrit. RBC cultures were established in 6-well plates and incubated at 37°C under 2% O_2_, 5% CO_2_, and 93% N_2_ atmospheric conditions as described (22). As shown in Fig. 1A, an increase in parasitemia to approximately 12% and 14% is observed after 24 and 48 hours of inoculation, respectively, followed by a decrease after 72 hours. Microscopic analysis of *in vitro* cultures showed singly and multiply infected cells with merozoites and ring stages of the parasite (Fig. 1B, arrows).

### Analysis of EV fractions isolated from iRBC supernatants

**Fig 2:**
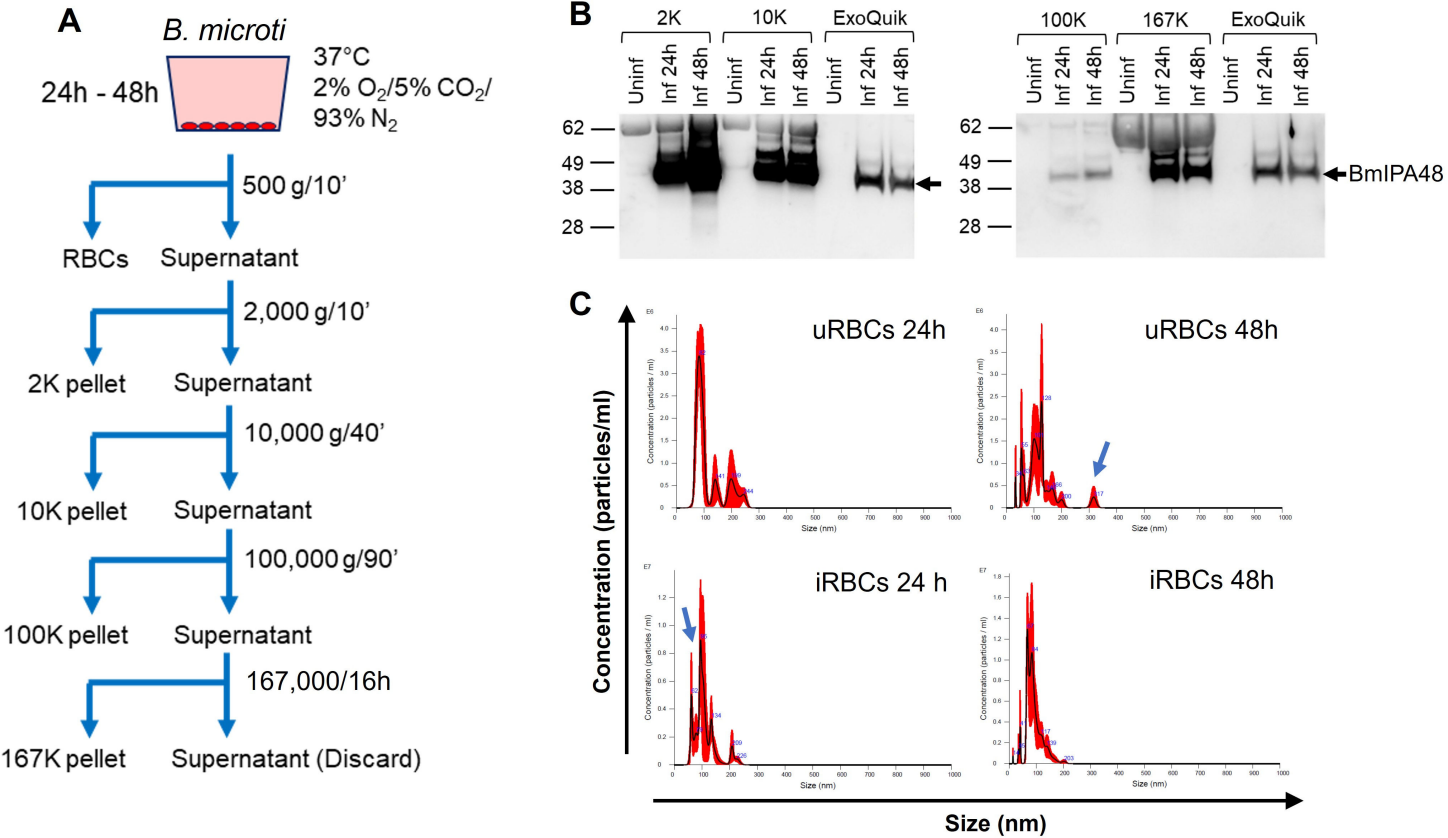
Analysis of EV fractions isolated from RBC culture supernatants. (A) Schematic for the isolation of EVs by sequential centrifugation. (B) BmIPA48 Western blots in EVs isolated from uRBC (Uninf) and iRBC (Inf) supernatants. (C) Size distribution of EVs isolated in 167K pellets by NTA. Arrows depict distinct EV populations observed in uninfected and infected samples.

To examine the release of EVs from *B. microti* iRBCs cultured *in vitro*, supernatants were collected at different times of infection and subjected to stepwise centrifugations (Fig. 2A). Western blots detected the parasite antigen BmIPA48 in EV pellets collected following centrifugations of 2,000 × *g* (2K), 10,000 × *g* (10K), 100,000 × *g* (100K), and 167,000 × *g* (167K) (Fig. 2B). The majority of BmIPA48 was found associated with EVs from 2K to 10K pellets, which represent large vesicles (>1 µm) and microvesicles (0.1–1 μm), respectively. The protein was detected to a significantly lesser degree in EVs from 100K pellets but markedly present in EVs from 167K fractions, which are expected to harbor exosome-sized vesicles (30–150 nm) (Fig. 2B).

Previous immunoelectron microscopy analyses of *B. microti*-infected RBCs and plasma from infected mice identified parasite proteins associated with vesicles of ~100 nm in diameter and tubular structures > 100 nm in length (12). Thus, we examined the size distribution of EVs found in our 167K fractions by NTA to determine if vesicles of similar characteristics were detected. As shown in Fig. 2C, the sizes of vesicles in uRBC and *Babesia* iRBC EVs isolated from 24 hour culture supernatants range from 60 to 250 nm. The mean diameter of uRBC EVs was 114.4 ± 15.9 nm, with peaks observed at 82, 141, 199, and 244 nm. *Babesia* iRBC EVs were similar in diameter to uRBC EVs, measuring 110.8 ± 1.8 nm; however, the size range included an additional smaller peak at 62 nm and peaks detected at 79, 95, 134, 209, and 226 nm (Fig. 2C). Interestingly, the mean concentration of EVs was twofold higher in 167K fractions of 24 hour iRBC cultures compared to uRBC cultures (3.45 × 10^8^ versus 1.53 × 10^8^ particles/mL, respectively). This difference increased fourfold after 48 hours of culture (1.16 × 10^8^ particles/mL in uRBC versus 5.03 × 10^8^ particles/mL in *Babesia* iRBC cultures). In addition, a widening in vesicle size ranges from 34 to 320 nm and from 14 to 203 nm was noticeable in 48 hour cultures of uRBCs and iRBCs, respectively. The highest concentrations of EVs in these 167K fractions were found in the 55–130 nm range for uRBCs and the 40–85 nm range for iRBCs (Fig. 2C).

### Assessment of macrophage uptake of EVs isolated from RBC culture supernatants and hamster plasma

**Fig 3:**
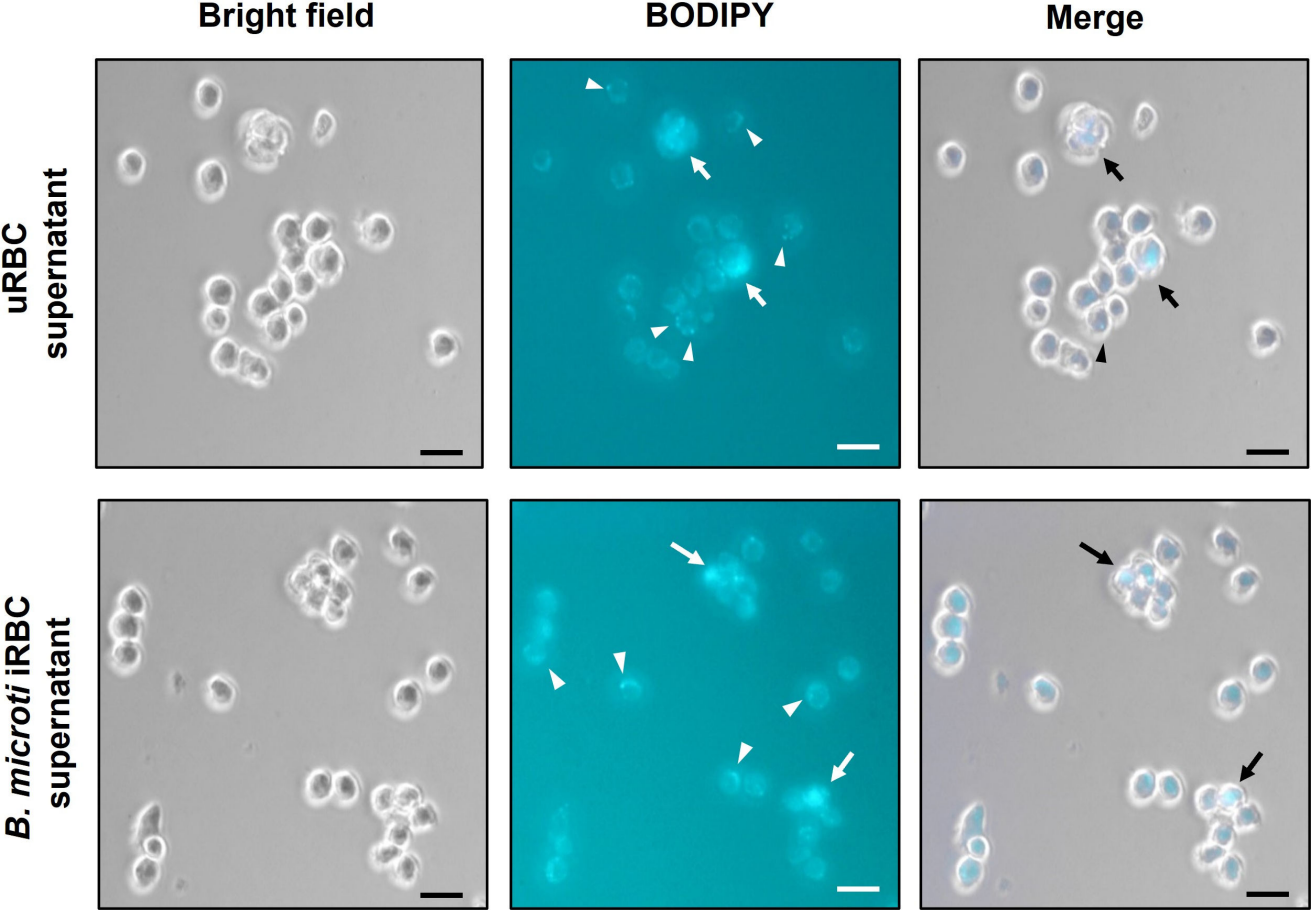
Assessment of macrophage uptake of EVs isolated from uRBC supernatants (A) and *B. microti* iRBC supernatants (B). EVs present in 167K fractions were labeled with BODIPY dye and incubated with monocyte-derived macrophages (ATCC TIB-202) for 90 minutes. Arrows show apparent internalization of BODIPY-labeled EVs. Arrowheads show localization of BODIPY-labeled EVs at the macrophage cell membranes. Bar, 15 µm.

To investigate whether macrophages were able to internalize EVs from *Babesia* iRBCs, monocyte-derived macrophages of the ATCC TIB-202 cell line were exposed to BODIPY-labeled vesicles present in the 167K fractions. As shown in Fig. 3, clusters of intracellular fluorescence and fluorescent punctate signals are observed in macrophages after 90 minutes of exposure to BODIPY-labeled EVs from uRBC and iRBC cultures. Fluorescent clusters were generally detected inside macrophages, suggesting internalization of BODIPY-labeled EVs (Fig. 3A and B, arrows), while punctate signals were primarily localized at the macrophage cell membranes (Fig. 3A and B, arrowheads). Exposure of cells to a dye-only control solution did not result in intracellular fluorescent staining (data not shown).

### Activation of NF-κB and alterations in cytokine secretion by EVs isolated from iRBC supernatants

**Fig 4:**
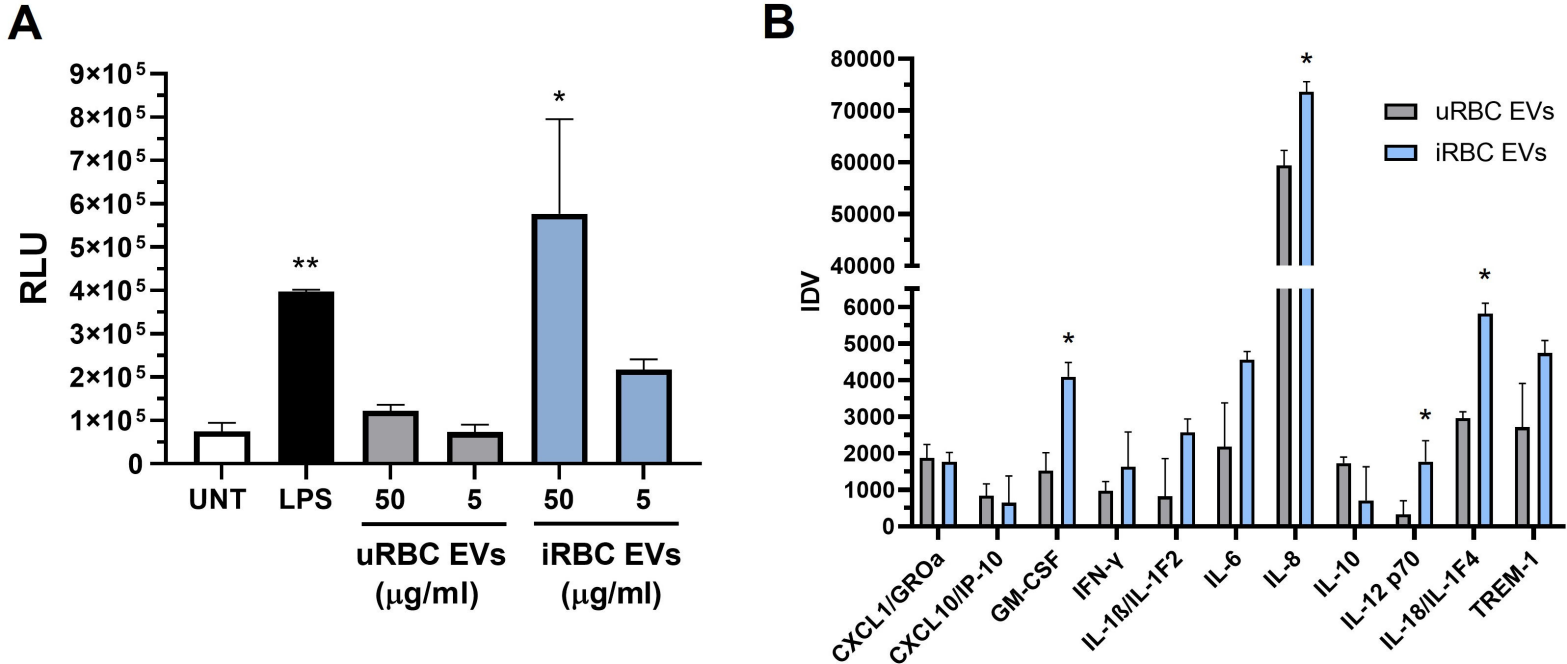
Macrophage NF-κB activity and cytokine production in response to EVs from *Babesia* iRBCs. (A) Luciferase activity was measured in macrophages treated for 24 hours with different protein concentrations of 167 K EV fractions isolated from uRBC or iRBC supernatants. UNT, untreated; LPS, macrophages treated with 5 µg/mL of LPS. Bars represent means + SEM of three experiments. **P < 0.01 compared to UNT; *P < 0.05 compared to 50 µg/mL of uRBC EVs. (B) Production of cytokines in macrophages treated with 50 µg/mL of uRBC (gray bars) or iRBC (blue bars) EVs for 24 hours. Culture supernatants were examined by the Proteome Profiler Antibody Array Kit (R&D Systems). Densitometric analysis of cytokine spots was performed using ImageJ software. Bars represent means + SEM of three experiments. *P < 0.05 compared to uRBC EVs.

The effect of EV treatment on the transcription factor NF-κB was examined in macrophages using luciferase reporter assays. Monocyte-derived macrophages (ATCC TIB-202-NFκB-LUC2) were incubated for 24 hours with different protein concentrations of EVs found in 167K fractions. As shown in Fig. 4A, a significant increase in NF-κB-dependent luciferase activity is detected in response to 50 µg/mL of EVs from *Babesia* iRBCs compared to uRBC controls. A modest increase was observed at the lower EV iRBC concentration of 5 µg/mL. In parallel with the increase in NF-κB activity, increases in pro-inflammatory mediators were detected in the supernatants of macrophages treated with 50 µg/mL of iRBC EVs compared to uRBC EVs (Fig. 4B). Among the specific cytokines known to be regulated by NF-κB (24), we found significant stimulation of GM-CSF, IL-8, IL-12, and IL-18 in response to treatment with iRBC EVs compared to uRBC EVs (*P* < 0.05).

## DISCUSSION

There has been a significant increase in EV research in the past decade, particularly on the role of EVs in host–pathogen interactions among diseases caused by protozoan parasites (25, 26). Silverman et al. reported the release of exosomes by *Leishmania* promastigotes in response to changes in temperature and pH (27). The uptake of *Leishmania* exosomes by macrophages stimulated IL-8 production (28). In Chagas disease, the release of EVs occurs in epimastigote and trypomastigote stages of *Trypanosoma cruzi*, causing the induction of pro-inflammatory cytokines and nitric oxide by macrophages (29). In *Plasmodium*, EVs are elevated in the plasma of malaria patients in proportion to disease severity (30). When purified from the plasma of malaria-infected mice, EVs induced potent activation of macrophages via Toll-like receptor (TLR) signaling (14). EVs released from *in vitro* cultures of *Plasmodium*-infected RBCs were shown to activate macrophages to produce cytokines and stimulate chemotaxis in neutrophils (16). In addition to playing a role in the pathology of malaria, *P. falciparum* takes advantage of RBC microvesicle pathways to induce the release of EVs from infected cells, mediating intercellular communication between RBCs, facilitating horizontal transfer of nucleic acids, and regulating parasite density and production of high numbers of gametocytes *in vitro* (31).

Prior work from Thekkiniath et al. (12) provided us with a blueprint to study the basis of EV release from parasitized RBCs. The authors suggested that *B. microti*-derived vesicles are actively exported during parasite development with little, if any, involvement from host–microvesicle pathways. Their findings were deduced from studies on EV pellets obtained by ultracentrifugation from murine plasma (12). Using *Babesia in vivo* models to study the biological activities of parasite-derived EVs carries certain limitations, given that EVs in plasma can originate from erythrocytes, leukocytes, platelets, and endothelial cells (32, 33). An additional caveat of working with vesicles isolated from plasma involves the capacity of EVs to carry cytokines, which could induce stimulatory activities in recipient cells (34). To minimize the effects of EVs from other cellular sources, the present study approached the isolation of EVs from *in vitro* cultured RBCs. Of note, a continuous *in vitro* model system for *B. microti* is currently unavailable; however, small-scale studies are possible using short-term cultures.

*B. microti* iRBCs cultured *in vitro* release EVs harboring the parasite antigen BmIPA48, as shown by ultracentrifugation of vesicle pellets from culture supernatants and immunoblot detection. The presence of BmIPA48 in vesicles was also detected through the enrichment of EVs from *B. microti* iRBC culture supernatants with the polymer-based reagent ExoQuick. Whether BmIPA48 is exported in close association with EV membranes or inside EVs remains to be determined. The latter scenario is more likely given the absence of a GPI-anchor motif or a transmembrane domain in BmIPA48 (35). A comparison of EV size distributions in 167K fractions of 24 hour uRBC and *Babesia* iRBC cultures showed notable differences, particularly among EVs < 100 nm in diameter. The detection of specific parasite-derived vesicles among these distinct EV populations, particularly tubular vesicles not previously observed in uninfected samples (12), will require further fractionation experiments using gradient centrifugation or size-exclusion chromatography. These approaches, combined with proteomics, will help us gain a better understanding of the protein composition and biogenesis of vesicles secreted from *Babesia* iRBCs to the extracellular environment. The finding that the overall concentrations of EVs in 167K pellets were higher as a result of infection has also been observed with *B. divergens* (13), suggesting that *Babesia* infection modulates microvesicle generation in RBCs as reported with *Plasmodium* (16).

Macrophage uptake of EVs was indistinguishable between vesicles isolated from uRBC and iRBC cultures. However, EVs isolated from iRBCs induced a significant increase in macrophage NF-κB activity and concomitant production of cytokines compared to EVs from uRBCs. Increases in pro-inflammatory cytokines are a hallmark of acute babesiosis in mouse studies (8, 9, 36, 37). The observed NF-κB and cytokine responses to iRBC EVs in macrophages warrant future studies to determine the extent to which *Babesia*-derived EVs participate in the innate immune response to infection (8).

**Fig 5:**
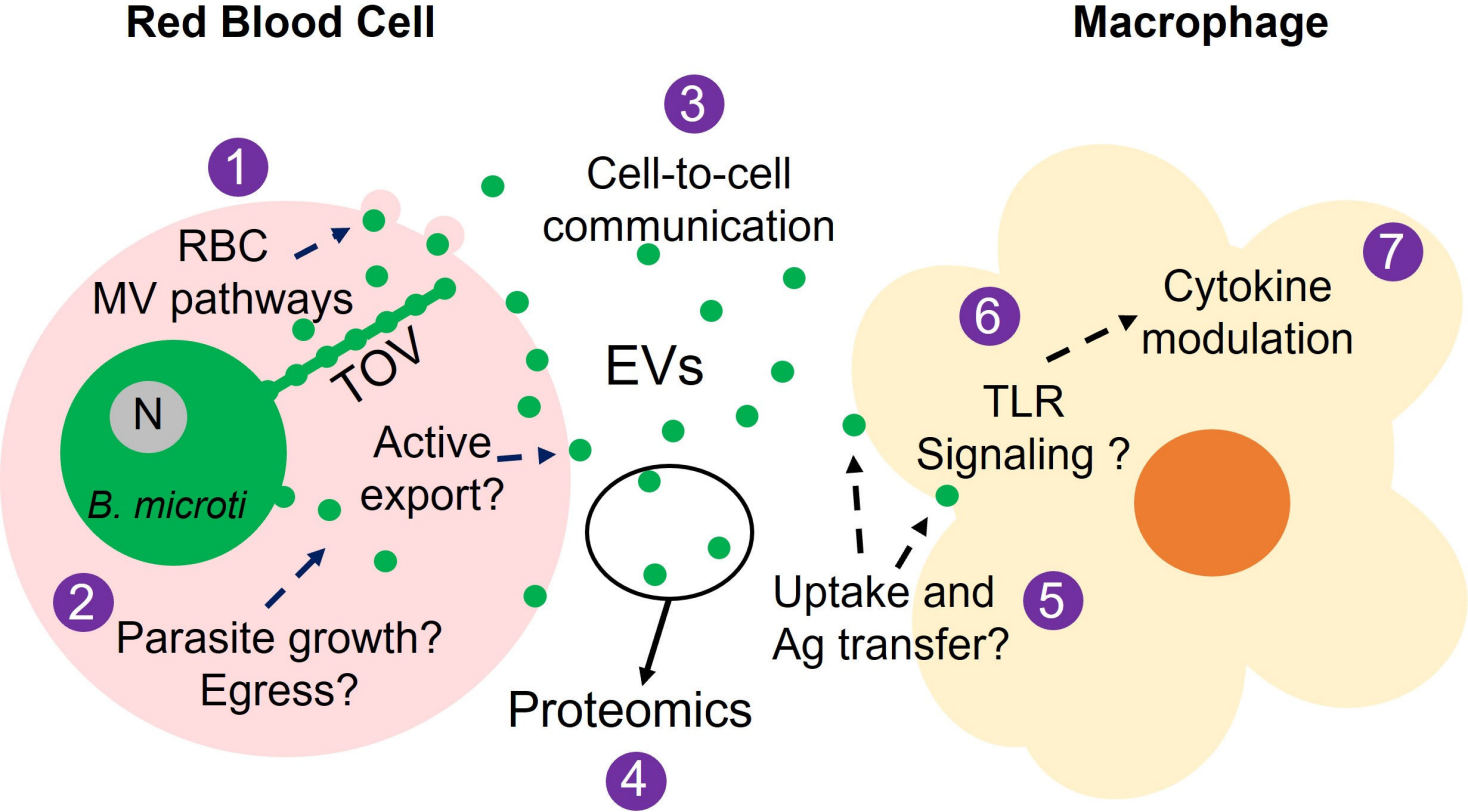
Plausible biological role of *B. microti* iRBC EVs in babesiosis. See text for details. Bm, *B. microti*; N, parasite nucleus; MV, microvesicle; TOV, tubes of vesicles (12).

In summary, the present study provides evidence supporting that *B. microti* infection induces the release of EVs from RBCs and that EVs released from parasite iRBCs cause phenotypic changes in macrophages. The identification of the TOV system as a protein export mechanism is a noteworthy discovery in *Babesia* biology; however, many questions remain that need to be addressed (Fig. 5): (i) Does *B. microti* manipulate RBC microvesicle pathways to induce the release of EVs to the extracellular environment? (ii) Is the extent of EV release dependent on parasite growth and egress? (iii) To what degree do parasite-derived EVs participate in cell-to-cell communication resulting in phenotypic changes of resting recipient cells? (iv) What are the parasite-derived protein cargoes encapsulated within EVs? (v) Are parasite antigens transferred from EVs to recipient cells, and if so, does this transfer result in changes in signal transduction pathways? (vi) Do parasite antigens enclosed within EVs engage macrophage Toll-like receptors (TLRs) and drive the cytokine response? (vii) How critical is the EV-dependent modulation of macrophage functions in the innate immune response to the parasite? Elucidating the roles of host and parasite factors involved in vesicle-mediated cell-to-cell communication and the modulation of host biological activities by *Babesia*-derived EVs will contribute to a better understanding of the mechanisms governing intracellular parasitism in babesiosis.
